# Suicidality and Non-Suicidal Self-Injury: A Narrative Review of Measurement, Risk, and Disparities among Minoritized and System-Involved Youth in the USA

**DOI:** 10.3390/children11040466

**Published:** 2024-04-14

**Authors:** Melissa L. Villodas

**Affiliations:** Department of Social Work, George Mason University, Fairfax, VA 22030, USA; mvilloda@gmu.edu

**Keywords:** youth, suicidality, mental health, non-suicidal self-injury, intersectionality, system involvement, positive youth development

## Abstract

Suicidality and non-suicidal self-injury (NSSI) among youth in the United States continue to be a growing and serious public health concern. With alarming rates of suicide trending in the wrong direction, researchers are committed to bending the curve of suicide and reducing rates by 2025. Understanding the antecedents and conditions, existing measures, and disparate prevalence rates across minoritized groups is imperative for developing effective strategies for meeting this goal. This study presents a narrative review of the operationalization, measurement, risk factors (e.g., firearms and social media), and disparities across race, ethnicity, age, gender identity, ability, sexual orientation, immigration statuses, and system involvement (e.g., foster care and juvenile justice) of suicidality and non-suicidal self-harm across youth in the United States. Implications for research, practice, and policy approaches that incorporate positive youth development, cultural, and youth participation in interventions are discussed.

## 1. Introduction

Suicide, the second leading cause of death among youth in the United States, has increased from 9.1% to 13% between 2000 and 2019 among people aged 10–34 [1,2]. In a national school-based survey of high school youth in the United States between the years 1997 and 2017 (N = 198,540), the overall prevalence rates of suicidal ideation, plans, attempts, and injury by attempt were 18.8%, 14.7%, 7.9%, and 2.5%, respectively [3]. Meaning, over the span of 16 years, almost 1 in 5 adolescents thought about suicide (18.8%), and more than 1 in 10 had a suicide plan (14.7%). This alarming trend is also seen among youth aged 10–14 as a U.S.-based study reported that suicide rates tripled from 2007 to 2017 [4]. Non-suicidal self-injury (NSSI) is also a growing concern with prevalence rates between 7.5 and 46.5% among youth across the U.S., with the onset of NSSI behaviors occurring at about age 12 [5]. Research on suicide and suicide risk during the COVID-19 pandemic gained attention becoming a notable public health concern as news reports on youth suicide referred to the pandemic as the “perfect storm” for stressors among children that increase the risk of suicide [6]. Hill and colleagues (2021) found that rates of suicidal thoughts and attempts were higher during some months of 2020 compared to the same months in 2019 among youth living in Texas and suspected that the months with higher rates corresponded with times when COVID-19-related stressors were heightened (e.g., stay-at-home orders and social distancing efforts) [7]. With alarming rates of suicide trending in the wrong direction, researchers are committed to bending the curve of suicide and reducing rates by 2025 [8]. Understanding antecedents and conditions, measures, and disparities around suicidality is imperative for developing effective strategies for meeting this goal. Therefore, this review offers a narrative summary of the operationalization, measurement, risk factors, and disparities across identity statuses and system involvement of suicidality and self-harm across youth in the United States. Information used to develop this narrative review was collected from the sources listed in Table 1.

### 1.1. Operationalization of Suicide

Suicide is a fatal self-injurious act with some evidence that the individual intended to die [9,10,11]. According to the fifth edition of the Diagnostic and Statistical Manual (DSM-5) of Mental Disorders, suicidal behavior or attempts [3,11,12] is defined as violent behaviors by an individual who at the time of the initiation of the behavior expected that the set of actions would lead to their death [13]. Suicidal ideation can be broken down into two categories. The first is active suicidal ideation, where an individual has thoughts about taking actions to end their life and has a plan and intent to act on it [11]. The second is passive suicidal ideation, in which an individual might have thoughts about death or wanting to be dead but has not identified a plan or intent to act [11]. Suicidal plans refer to the formulation of a specific method through which the individual intends to die [14]. The word suicidality is often used to encompass suicidal ideation, plans, attempts, and behaviors [15,16].

Suicidal ideation and suicide plans are key risk factors for engagement in suicidal behaviors [3]. During the transition from childhood to adolescence, suicidal thoughts and behaviors are documented to increase with most youth who transition from suicidal thoughts to suicidal behaviors doing so within 1–2 years after the onset of suicide ideation [14,17]. Therefore, in the study of suicide among youth, it is critical to examine ideation and plans of suicide in addition to attempts and behaviors.

### 1.2. Operationalization of Non-Suicidal Self-Injury

Non-suicidal self-injury (NSSI) differs from suicidal behavior and attempt in terms of motivation and does not fall under the term suicidality [11]. NSSI is described as the act of repeatedly inflicting shallow, yet painful, injuries to the surface of the body with the intent to reduce negative emotions or to resolve an interpersonal difficulty but with no intent to die [11,13]. NSSI behaviors may include cutting or scratching one’s wrists or arms, self-hitting, banging one’s head against the wall, or burning skin, among others [5,11,18]. It is hypothesized that NSSI is a result of an individual being unable to regulate overwhelming emotions and becomes a form of avoidance or provides feelings of temporary relief [19,20]. Self-harm is defined as any kind of self-injurious behavior (including suicide and NSSI). The term self-harm becomes un-useful when it is the only word used to describe a behavior. Using the word self-harm does not allow researchers or practitioners to disaggregate the intent of the behavior [11]. Therefore, this review uses terms that fall under the scope of suicidality or NSSI to provide clarity when addressing research that discusses the intent of self-harm.

NSSI is important to assess alongside suicidality. Though NSSI is distinct from suicidal behavior due to intent, suicide attempts and ideation have been found in clinical and non-clinical samples of youth in studies on NSSI [5,21,22]. NSSI has also been associated with several health risk behaviors that impact mental health, like substance abuse, risky sexual behaviors, and maladaptive eating habits [5].

### 1.3. Measurement of Suicidality and NSSI

In a 2015 systematic review, Ghasmei and colleagues identified 21 scales for assessing suicidal ideation [23]. They determined there is no gold-standard approach to study suicide-related ideation. Of the 21 scales identified, 9 of the scales targeted youth (see Table 2). Apart from the scales extracted in the 2015 systematic review, questions that assess suicidal thoughts and behaviors are often binary with yes or no options or count data and include questions like (1) “During the past 12 months, did you ever seriously consider attempting suicide?” (suicidal ideation); (2) “During the past 12 months, did you make a plan about how you would attempt suicide?” (suicidal plan); (3) “During the past 12 months, how many times did you actually attempt suicide?”; and (4) “Did any attempt result in an injury, poisoning, or overdose that had to be treated by a doctor or nurse?” (injury by attempt). These questions have been documented to offer substantial reliability in previous studies and ask about several of the thoughts and behaviors associated with the act of suicide [3]. Examining each aspect in the investigation of the prevalence and incidence of suicidal behaviors and ideation as well as the existence of a plan is critical to understanding potentially lifesaving intervention targets.

NSSI did not have its own clinical diagnosis before its proposed addition in the publishing of the DSM-5. Before then, assessments were not standardized and varied widely. Some assessments focused on whether non-suicidal self-injury (NSSI) was present or absent, while others delved deeper into assessing various aspects of NSSI, such as how often it occurred, the situations in which it occurred, which parts of the body were injured, and the likelihood of these behaviors continuing over time [24]. In response to changes in the DSM-5, three assessments have been developed to measure diagnostic criteria for NSSI which are also listed in Table 2 [24].

Finally, for individuals that complete suicide, the cause of death most often involves a determination as such by medical examiners and coroners [25] and serves as a key data point for understanding its prevalence across populations. However, due to the stigma around this classification and, at times, a lack of evidence around intent, it is likely that suicides may be underreported [25]. The second-order challenges that came with the COVID-19 pandemic, like increases in suicidality, prompted attention to be directed to improved data collection systems that identify changes in rates of completed suicides and suicide attempts in near real time [26,27]. Challenges continue to abound, however, in capturing this data accurately across children and youth and especially so across minoritized and system-involved populations of youth.

## 2. Antecedents and Conditions of Suicidality and NSSI among Youth

### 2.1. Mental Illness and Substance Use

The adolescent stage of life is marked by transitions and changes in several aspects of life including building an identity, developing self-esteem, increasing independence and responsibility, and building new relationships [9]. This time in life is a vulnerable period associated with the onset of mental health challenges as psychosocial events unique to adolescence can become challenging with the concurrently rapid and substantial developmental changes that occur in the brain and in hormones [28]. A recent report by Mental Health America states that young people aged 11–17 struggle with mental health more than any other group, with anxiety and depression among the most common psychiatric illnesses affecting youth [28,29]. One in every four to five youths in the United States meets the criteria for a mental disorder that is associated with severe impairment or distress [30]. The onset of mental illness in adolescence is impactful beyond adolescent years with 50% of adult mental health disorders occurring by the age of 14 [30].

Mental health challenges like substance abuse, anxiety disorders, depressive disorders, and psychological distress have been identified as antecedents of suicidality and NSSI in young people and NSSI [30,31,32,33]. Depression has been consistently observed as one of the most significant risk factors for suicidal behaviors and ideation, with criteria for depression having been found in 50–60% of suicide cases [9,34]. Similarly, findings from a 2015 systematic review found that post-traumatic stress disorder (PTSD) is associated with elevated levels of suicidal ideation in youth ranging from 30 to 80% and suicide attempts ranging from 15 to 50% [35]. Additionally, regardless of the type of substance used, youth who use substances including cigarettes, alcohol, ketamine, and methylenedioxymethamphetamine (MDMA) have been found to have statistically significant higher rates of suicidal ideation and attempts compared to youth who do not use these substances, with alcohol abuse and dependence being most strongly associated with suicide attempts among youth who use substances [36,37].

Together, PTSD and depression have been found to be a stronger predictor of suicidal behavior and thoughts than they would be on their own [34]. Similar assertions have been made related to comorbidity with depression and anxiety; however, anxiety as a risk factor for suicidal ideation and behaviors among youth apart from the co-occurring presence of depression is not well studied or understood [38]. This has also been the case for substance use and suicide as the association between substance use and suicidal behaviors is reduced when controlling for depression in statistical tests [37].

NSSI has also been linked to mental health disorders. Comorbidity is commonly reported with borderline personality disorder and eating disorders and with NSSI also being linked to other disorders including PTSD, obsessive–compulsive disorder (OCD), conduct disorders, mood disorders, and anxiety disorders [5]. There are several other antecedents to NSSI beyond mental health challenges. In a 2017 systematic review by Cipriano and colleagues, they discuss that potential etiologic factors of NSSI include childhood maltreatment, insecure parental and maternal attachment, neglect, and stressful life experiences among those with poor emotional regulation.

### 2.2. Intrapersonal, Interpersonal, and Community Factors

Social isolation, lack of friendships, relationship problems, and interpersonal trauma are additional conditions shown to be associated with increased youth suicidal behaviors [2,39]. Furthermore, previous suicide attempts, personality characteristics (e.g., impulsivity, poor problem-solving skills), family factors (e.g., parent suicide, absence of communication, neglect, violence at home, etc.), life events (e.g., interpersonal losses, peer rejection, bullying), contagion imitation (e.g., repeating NSSI or behaviors related to suicidality as a result of highly publicized coverage of the behavior), and availability of means (e.g., access to firearms) have also been identified as antecedents and conditions of youth suicidal behavior [9,40].

### 2.3. Social Media Use and Cyberbullying

In 2022, 97% of youth aged 13 to 17 reported they use the internet daily, with the top social media platforms being YouTube, TikTok, Instagram, and Snapchat [41]. Across studies in Ontario, the United States, and the United Kingdom, daily social media use of two hours or more and heavy use of electronic devices (5+ hours per day) was associated with significant increases in the odds of suicidality compared to youth in this study who use social media and electronic devices for less time [42,43]. A 2021 systematic review on suicidal thoughts and behaviors by Macrynikola and colleagues concluded that cyberbullying has been identified as a mediating factor in the relationship between social media use and suicidality [44,45,46]. One study using data from the CDC Youth Risk Behavior Survey on high school students found that 15% of high schoolers who were cyberbullied reported making a suicide attempt compared to 5% of high schoolers who were not cyberbullied [47]. Cyberbullies themselves are also found to be at a greater risk of suicidality compared to those who are not cyberbullies, but this risk is still less pronounced than those who experience cyberbullying directly as these victims are at a greater risk of self–harm and suicidal behaviors [48].

### 2.4. Access to Firearms

Access to firearms is another important consideration to hone in on. Firearms are currently the leading cause of death in children and youth aged 0 to 14 in the United States and one of the leading means of youth suicide (see Table 3) [49]. From the years 2010 to 2020, 42% of suicide deaths in the United States among children and youth aged 10 to 19 years were caused by firearms [50]. A national study of youth in the United States indicates that one-third of youth live in a home with a firearm, with 41% reporting they have easy access to the firearm [51]. A study by Johnson and colleagues (2010) indicates that 75% of deaths by suicide due to firearms were completed with the firearms of the youth’s parents and usually occurred within the home [52].

## 3. Rates Suicidality and NSSI among Youth

Trends indicate disparities in suicidality are present among youth of color (i.e., Hispanic or non-White youth) compared to White youth generally; therefore, it is important to highlight prevalence rates that are specific to racial and ethnic groups as specific factors unique to these groups and their intersectional identities exist [34]. A summary of the discussion below can be found in Table 3.

Non-Hispanic American Indian or Alaskan Natives (AI/ANs) who make up just 1.3% of the U.S. population have the highest rate of suicidal thoughts and plans as well as NSSI compared to all other documented racial and ethnic groups among both youth and adults [3,55,56]. Youth made up 9.8% of suicides among the AI/AN population between 2003 and 2014 [57]. In 2015, the AI/AN suicide rates for AI/AN boys were 26.22 per 100,000 and 12.21 per 100,000 for girls [3]. Suicide among AI/ANs is the number two cause of death among 10–24-year-olds, with suffocation being the primary cause of death [53]. Suicide among AI/ANs is documented to peak during adolescence and young adulthood and then decline [56]. In a 2018 study, Monto and colleagues [54] found that 20.79 per 100,000 youths who identified as AI/AN reported NSSI in the previous 12 months.

Non-Hispanic White boys and girls follow AI/AN youth in rates of suicide [3]. In 2017, suicide rates were 13.19 per 100,000 among White boys and 2.87 per 100,000 among White girls. Among high school youth, White youth are similar to the overall U.S. population across considering a suicide attempt but have smaller percentages of planning and attempting suicide [56]. Suicide among non-Hispanic White youth is the number two cause of death among 10–24-year-olds with suffocation being the primary cause of death among 10–14-year-olds and firearms being the primary cause of death among 15–24-year-olds [53]. Non-Hispanic White youth were the third highest group (17.71 per 100,000) in a pooled sample of youth to report NSSI in the last 12 months [54].

Following White youth, non-Hispanic Black boys and girls have the next highest rate of suicide but the lowest rate of NSSI [3]. In 2017, rates of suicide among Black boys were 7.33 per 100,000, and among Black girls, rates were 2.65 per 100,000 [3]. Rates of NSSI in 2015 were 12.10 per 100,000 [54]. According to a 2019 study by Lindsey and colleagues, Black youth were the only group that had increases in rates of suicide attempts between 1991 and 2017. Though rates of suicide among Black youth in 2017 were less than AI/AN and non-Hispanic White youth, trends do not appear to be moving in the same direction for Black youth [3]. In 2019, suicide was the fourth leading cause of death among Black youth aged 10–14 years old with suffocation as the primary cause of death and was the third leading cause of death among Black youth aged 15–24 with firearms being the primary cause of death [53]. This is especially so among young Black males aged 10–19 as proportions of suicide involving firearms increased at a faster pace than other racial groups [58].

Hispanic youth follow next in the rate of suicide with suicide among Hispanic boys at 7.24 per 100,000 and girls at 2.84 per 100,000 in 2017 [3]. In 2019, suicide was the third leading cause of death among Hispanic youth aged 10–14 years old and the second leading cause of death among youth aged 15–24 with suffocation as the primary cause of death [53]. In 2015, Hispanic youth had the second highest rate of NSSI among a pooled sample of youth at 19.19 per 100,000 [54].

Non-Hispanic Asian American or Pacific Islander (AAPI) youth have the lowest documented suicide rate in the United States compared to all other races and ethnicities [3]. Rates among AAPI boys were 6.74 per 100,000 and 3.63 among girls in 2017. In 2019, suicide was the number three cause of death among 10–14-year-olds and the number one cause of death among 15–24-year-old AAPI youth with suffocation cited as the primary cause of death [53]. AAPI youth had a rate of 14.98 per 100,000 for NSSI in 2015, which was just higher than Black youth [54].

Suicide rates alone only provide a snapshot of the problem. To recognize the disparities that youth of color face across and within the spectrum of race and ethnicity, it is critical to explore suicidality and NSSI in the study of suicide as these are critical points of intervention to decrease the rates of self-harm cited above. Though death by suicide is tragic and worth addressing among every racial and ethnic group, I offer a discussion on what these rates and trends mean for two groups specifically: Black youth and American Indian and Alaskan Native youth.

### 3.1. Changing Trends in Suicide among Black Youth

In 2019, Lindsey and colleagues found that between 1991 and 2017, youth who identified as White, Hispanic, AAPI, and AI/AN all had significant linear decreases in self-reported suicide attempts. Black youth, however, had a significantly linear increase in self-reported suicide attempts with a significant linear increase for Black boys and an alarming rate of acceleration among Black girls [3]. In the same 2019 study, there were significant linear decreases in both suicidal ideation and suicide plans across the years among all subgroups of youth except for nonsignificant trends found for Black girls and AI/AN boys for suicide plans, as well as AI/AN girls and non-Hispanic multiple-race youth of either gender for suicidal ideation and plans [3]. Furthermore, a study by Sheftall and colleagues (2022) found that from 2003 to 2017, both Black boys and girls experienced a significant upward trend in suicide rates with Black youth aged 15 to 17 showing the largest increase [39]. By examining racial and ethnic differences across suicidality and suicide rates, we are able to see a bigger picture of increasing trends in Black youth suicidality which has caused researchers to “Ring the Alarm” on Black youth suicide [59,60].

There are a variety of explanations for differences in outcomes among Black youth. Some explanations include the misclassification of mental health challenges, the under-reporting of suicidal ideation in mental health treatment due to mistrust, cultural influences that teach youth to be tough and to hide their emotions, and the compounding effects of ACEs, poverty, and racism that often burden Black youth [2,61]. Further, research has shown that suicide attempts among both Black and Hispanic youth living in urban areas occur at almost twice the national rate which may suggest that where an individual lives and the stressors of that lived environment may contribute to the exacerbation of mental health challenges that lead to suicidality [62].

### 3.2. Suicide Rates with American Indian and Alaskan Native Youth

Like the explicated discussion on Black youth, a discussion about the alarming rates of AI/AN youth over the years requires further elaboration as well. In a 2018 study of risk and protective factors of suicidal ideation in one AI community, it was found that depression was one of the strongest risk factors for suicidal ideation, consistent with the previous literature on suicidality and youth generally [63]. The authors discussed that the etiology of depression may be rooted in cultural traumas, adverse childhood experiences, and socioeconomic disadvantages that may serve as stressors for these youth [63]. Another study discussed suicidality being linked to the breakdown of intergenerational mentorship and support as a result of both historical and contemporary colonialism with these groups [64]. Substance abuse has been documented as a particularly concerning contributor to NSSI, suicidality, and suicide among AI/AN as substance dependence and abuse start at younger ages and are higher among AI/AN compared to other races and ethnicities in the United States [65].

## 4. Suicidality, NSSI, and Intersecting Identities of Youth

In addition to racial and ethnic disparities and changes in trends, there are also disparities related to intersectional identities of gender, sexual orientation, disability, and immigration that offer deeper but not fully sufficient insight into the prevalence and incidence of suicidality and NSSI.

### 4.1. Gender

Female–male differences have been reported for suicidal behaviors with males documented to experience higher mortality from suicide and females documented to experience higher suicidal ideation and nonfatal suicide attempts [12,34]. Research on gender differences outside of the binary female–male categories reports that transgender adolescents disproportionately report higher suicide attempts compared to cisgender (personal identity corresponding with the sex assigned at birth) youth [66]. In a 2018 study by Toomey and colleagues on adolescents ages 11 to 19, female-to-male youth reported the highest rate of suicide attempts (50.8%), followed by non-binary transgender youth (41.8%), male-to-female youth (29.9%), questioning youth (27.9%), cisgender female youth (17.6%), and cisgender male youth (9.8%) [66]. When considering how gender identity intersects with race and ethnicity, one study found that within their sample, race was only a significant predictor of suicide attempt with AI/AN transgender youth and noted how these two identities that already experience high rates of suicidality intersect to create greater risk [67]. Prevalence rates in the United States on death by suicide are not present across these gender identities as autopsy reports do not collect information on gender beyond male and female identities [66]. This limits the ability of researchers to understand trends more robustly across gender identities and other intersectional identities.

Regarding NSSI, a 2019 study by Taliaferro and colleagues found that transgender youth who were assigned female at birth were more likely to report NSSI than transgender youth who were assigned male at birth. They also found that this is consistent across studies as youth who are assigned female at birth demonstrate a greater prevalence of NSSI whether they are cisgender or transgender [68].

### 4.2. Sexual Orientation

In the Toomey and colleagues study mentioned above, researchers found that non-heterosexual sexual orientation was associated with higher odds of suicidal behavior, which is consistent with the extant research that sexual minority youth have shown higher risks of suicidal ideation and suicide attempts from adolescence to adulthood compared to their heterosexual counterparts [66,69]. In a 2007 study of high school youth, although lesbian, gay, and bisexual (LGB) youth made up 7% of the sample size, LGB youth accounted for 67% of NSSI in the study, highlighting their disproportionally high experiences with self-harm behaviors (Reinert et al., 2014) [70]. Among LGB youth aged 12–14, studies indicate that this age range and sexual minority status accounted for 24% of deaths by suicide between 2013 and 2015 [69,71]. In a 2020 study by Giano and colleagues, they examined risk profiles using latent profile analysis to identify risk factors of suicidal behavior among a representative sample of LGB youth in public high schools in the United States [69]. In the highest-risk profile—characterized by elevated mean scores in alcohol consumption, bullying, poor grades, low sleep, and electronics use—identifying as LGB and female was associated with a 52% increase in suicide attempts while identifying as LGB and Black was associated with increased chances of suicide by 40% [69].

Age is an important factor to consider among sexual minorities and research on suicidality. In the study by Giano and colleagues [69], each yearly increase in age was associated with a 14% decrease in the likelihood of suicide attempts, which draws attention to young LGB youth. Though asexual youth are not often studied within the scope of sexual minority youth, a study by McInroy and colleagues (2020) found asexual youth 14–19 years old were less likely to attempt suicide than their non-asexual peers [72].

### 4.3. Immigrant Youth

Regarding suicidality among foreign-born immigrant youth, most of the research in this area in the United States has focused on Mexican youth, who represent the largest immigrant group. Unfortunately, this often excludes many other immigrant groups living in the United States [73]. One 2006 study by Borges and colleagues examined differences in suicidal ideation and self-injury in a more diverse group of immigrant youth from China, Haiti, Brazil, Jamaica, Vietnam, and the Dominican Republic. They found that there were no differences in the risk of self-injury or suicidal ideation based on the immigration status of high school-aged youth; however, they could not disaggregate these results due to the small sample size [73]. Among Mexican youth specifically, a 2018 study found that being born outside the United States was a risk factor for suicide to which the writers posited that this may be due to limited socioeconomic opportunities [74]. This conflicts with research among Hispanic youth broadly that indicates being born outside the United States is a protective factor [74,75]. Similar findings on the protective factor of immigration status have also been found among Asian youth who are immigrants in the United States [76].

### 4.4. Youth with Disabilities

Disability, which refers to physical or mental impairments that limit an individual’s functioning or ability to engage in age-appropriate tasks and some life activities, is another intersectional identity to consider [77]. Disabilities may include emotional and mental health problems, autism spectrum disorder (ASD), hearing impairment, vision impairment, physical disability, learning disability, and speech and language problems, among others. Though mental health problems are often discussed in research on suicidality and NSSI, the other disabilities listed have varying degrees of consideration. For example, in an investigation on suicide attempts among youth with self-reported disabilities, Moses (2017) found that youth with disabilities (YWD) in the study sample were 3.6 times more likely to report a suicide attempt in the last year compared to their peers and that having more than one disability triples the risk of suicide attempts [77]. Following youth with mental and emotional health disabilities, youth with autism spectrum disorder were most likely to make multiple suicide attempts and youth with hearing and vision impairments were more likely to report suicide attempts than youth with learning disabilities [77].

Overall, the prevalence of NSSI among youth with intellectual disabilities has been found to be higher compared to youth with sensory impairment [78]. Coupled with sexual minority identification, the emerging literature is currently mixed in the interaction between sexual identity and disability status on suicidality [79,80]. Additionally, there is more to be learned about the interaction between disability status and racial and ethnic identity related to suicidality and NSSI.

### 4.5. System-Involved Youth

Finally, it is imperative to discuss groups of youth who are involved in child welfare systems like foster care and the juvenile justice system as the youths in these systems represent an important boundaried population where the risk of suicide is of great concern [81]. While it is natural to assume a greater risk of suicidality and NSSI among system-involved youth due to the challenges that come with the conditions that contribute to system involvement, few studies have examined the prevalence of suicidality among system-involved children and youth compared to those who are not in systems of care [82]. For example, in a 2017 systematic review and meta-analysis by Evans and colleagues that compared suicidality in children and youth in care and non-care populations, just five comparative studies met the criteria. While research is limited, discussed below is a summary of what is known about suicidality and system involvement.

#### 4.5.1. Foster Care System

In a 2006 study by Pilowsky and Wu, they found that youth aged 12 to 17 with a history of foster care placement were four times more likely than those without a history of foster care placement to have reported suicide attempts in the last 12 months [83]. A more recent study examining suicide and child protective system (CPS) involvement in California from 1999 to 2017 revealed that children and youth who were placed in foster care had almost 5 times the odds of suicide compared to children without any CPS involvement and those with any CPS involvement had 3.6 times high odds of suicide than those without CPS involvement [84]. Explanations for this increased risk can be attributed to many of the antecedents to suicidality mentioned above, including reduced social support, traumatic experiences, maltreatment, and mental health challenges that are exacerbated among system-involved youth [85,86,87].

#### 4.5.2. Juvenile Justice System

Suicide is a leading cause of death among justice-involved youth and rates of suicidality and NSSI among youth involved in the juvenile justice system also appear to be elevated compared to the general population [88]. A 2015 study of suicidality among youth in the juvenile justice system by Stokes and colleagues examined the prevalence of suicidality among delinquent youth living in the community, youth who were incarcerated, and at multiple points of contact [89]. Past-month suicidal ideation was found to be higher in post-adjudicated youth than in pre-adjudicated youth [89,90,91,92,93]. Prevalence rates among youth who were assessed during detention were high with the rate at 52% (past two weeks) and 25% (past 6 months among justice-involved girls) [94,95]. Explanations for the risk among justice-involved youth, like the general population, include a history of mental health challenges (youth in the juvenile justice system experience mental health problems at 2.3 times more than those without such involvement), trauma, and sexual abuse, feelings of hopelessness and isolation, family stressors, and psychosocial stressors [89,91,96].

## 5. Discussion

Suicidality and NSSI are issues that require expedient attention as the decision to act on this public health issue is a matter of life and death. Some antecedents and conditions of youth suicide that were identified included the misclassification of mental illness, stigma related to mental health services, and poor access to mental health services, particularly among youth of color. The mental health considerations of antecedents and conditions around suicidality and NSSI point to the importance of exploring the presence of adverse childhood experiences (ACEs) in the assessment of children and youth in medical settings as well as other settings youth frequent like schools. Assessment in these settings is particularly important as access to mental health services may be explored only when internalizing and externalizing symptoms of mental health challenges finally emerge. With increased assessment around ACEs and co-morbid mental health conditions, early intervention prevention opportunities may be identified. Additionally, evidence around access to firearms in addition to social media use and cyberbullying were discussed in this review. Recommendations from the Center for Gun Violence Solutions at Johns Hopkins University (2023) to decrease suicidality and NSSI through firearms include policies that address access to lethal means for youth, otherwise known as child access prevention laws (CAP laws) that create criminal penalties for the firearm owner if a child accesses their unsecured gun [60]. Their report indicates states that have implemented strong CAP laws have 41% lower rates of firearm injuries among minors compared to states that had weak or no CAP laws [60]. This review also examined vulnerable identities as evidenced by the emerging literature about the prevalence and incidence of suicidality and NSSI and included a summary of immigration status, disability status, and other intersectional identities. Though there are notable gaps in knowledge and mixed findings around intersectional identities comprising race, ethnicity, gender, sexual orientation, immigration, and disability, the interaction of two or more minoritized identities creates a greater risk for suicidal ideation and behaviors. Testing these intersections of identity is consistent with Black feminist thought around the term “double jeopardy” introduced by Francis Beale in 1972 [97]. Intersections of the identities discussed might otherwise be referred to as triple, quadruple, or simply multiple jeopardy to account for compounding multiple identities that lead to discrimination or oppression and likely exacerbate one’s risk for outcomes like suicidality and NSSI [97].

The antecedents and conditions of suicidality and NSSI are layered and complex and require creative approaches to support the mental health of youth, particularly so among vulnerable groups. Culturally responsive and positive youth development-oriented approaches may be promising avenues to explore. A systematic review by Shahram and colleagues (2021) highlights protective factors that may potentially contribute to resilience against suicidality among youth, with age, sex and gender, and Indigenous identity as important intersecting considerations [98]. These factors include positive self-appraisal (e.g., pride, self-esteem, and cultural identity), a “zest for life” (e.g., life satisfaction, life-oriented beliefs, internalized motivational drive for achievement, and gratitude), coping skills (e.g., emotional intelligence, capacity to share emotions, and self-forgiveness), social support systems (e.g., school, friend, community, family, and parent connectedness), and inclusive environments (e.g., social acceptance, community, neighborhood and school safety, and freedom from discrimination) [98]. This synthesis of research on resiliency aids in the development of future research on how to promote resilience at the community and system levels in addition to intra- and interpersonal levels.

### 5.1. Limitations

This narrative review provides a summary of research on minoritized and system-involved youth suicidality and NSSI. While a summary of this topic is necessary and important for understanding the vulnerability of these populations of youth situated alongside the prevalence and incidence of suicidality and NSSI in the United States nationally, it is important to note the limitations associated with this review. Narrative reviews are guided by a topic of interest and have no specified search strategy [99]. As such, this narrative review is not systematic and followed no specified protocol to guide the review. That said, systematic reviews are referenced throughout the review on many of the topics discussed and, therefore, the intention of the narrative review is to situate the findings within a greater narrative of vulnerability by topics of minoritized and intersectional identities and system-involved youth. Furthermore, another limitation is that there are likely minoritized populations of youth that are not explored in this review. This may include unhoused youth, youth involved in sex trafficking, and religious minorities, among others who may experience vulnerability. Despite the limitations that come with a narrative review, there is value to the accessibility of this topic for the purposes of learning and considering youth suicidality and NSSI more broadly.

### 5.2. Implications for Research, Policy, and Practice

With the information presented in this narrative review, it is important to consider implications for research, policy, and practice. Research, policy, and practice recommendations have been made to understand antecedents and conditions of suicidality specific to Black youth [59]. These recommendations include (1) increased funding and attention by the National Institutes of Health and Mental Health; (2) demonstration projects that provide opportunities to test, assess, and advance promising practices; (3) the promotion of existing best practices; (4) community engagement and awareness that includes clergy, elected officials, and fraternal and civic organizations; (5) a national website and repository for data on suicidal behavior; and (6) engagement of state and local governments [59].

Additionally, implications for future research among AI/AN youth often involve prevention programs and efforts to minimize substance abuse and calls for exploratory research have been made to design effective and culturally congruent screening and prevention approaches to minimize the burden of substance abuse and suicidality among AI/AN youth [100]. One existing intervention includes ‘Qungasvik’, which has been tested with Alaskan Native Youth [101]. While the testing of Qungasvik produced promising results for the prevention of suicide risk in these rural Alaskan Native communities, the intervention did not produce similar results for alcohol risk [101]. This underscores the continued need for identifying or developing interventions that address co-occurring risk. To that end, much can be learned from positive youth development approaches with this population. One intervention with Indigenous Alaskan youth includes digital storytelling, which was found to be a useful tool in suicide prevention [102]. As part of this intervention, youth participants created hope kits which were thought to facilitate stronger, protective intergenerational bonds and were used as a reminder of key reasons for living [102].

Research on youth with marginalized and intersectional identities is scarce and when considering the compounding burden of multiple jeopardy we see that youth with intersectional identities are at risk of suicidality and NSSI. When system involvement is introduced, the risk is greatly elevated. Mental health treatment and the establishment of formal and informal infrastructures that build responsiveness to suicidality among system-involved youth and those with multiple intersecting identities is an area of research that requires increased attention.

Future research studies should also prioritize disaggregating immigration status and country of origin in the investigation of suicidality and NSSI and explore trends over time as studies suggest the strength of the risk or protective factors related to immigration across ethnic backgrounds may be moderated by age [74]. Disaggregating immigration status, race, ethnicity, and other features of populations is important for strengthening approaches to address suicidality and NSSI [103]. This consideration of culture and lived experiences makes it possible to understand the needs, disparities, and opportunities to better support specific groups and achieve health equity for all [103]. Furthermore, it is likely that there is much to be learned about the prevalence and incidence of suicidality and NSSI among undocumented immigrant youth who may experience challenges that are not captured in research due to their immigration status. Researchers should consider the use of administrative data through medical offices and hospitals that serve these young people in service to understand the prevalence and incidence of suicidality and NSSI to better assist youth who experience this.

To increase exposure to the types of help available for children and families, researchers and mental health practitioners should partner with communities to develop psychoeducation materials on suicidality and NSSI that can be widely disseminated across spaces youth and families encounter. For example, supermarkets, schools, community centers, and churches become important partners for building awareness on how to seek help and support. Through partnering with community organizations and businesses such as these, information aimed at decreasing stigma and building awareness of mental health resources can build lifesaving connections to help youth experiencing suicidality. Considerations should also be made about the availability of information in multiple languages as well as accessibility aids that may assist individuals with varied reading comprehension levels or visual and auditory impairments.

Furthermore, opportunities for research abound in partnering with young people themselves to both understand conditions by which they feel safe to disclose suicidal thoughts and also to engage them in leadership opportunities that aid in prevention. For example, Hawai‘i’s Caring Communities Initiative (HCCI) for Youth Suicide Prevention is an early intervention approach to youth suicide prevention that uses a youth leadership model focused on youth empowerment, relationship, and team-building activities, suicide prevention training, and community awareness events [104]. The youth leaders on HCCI were also trained in evidence-based suicide prevention strategies that focus on producing public messages about suicide in a way that is unlikely to increase the risk of suicidality for vulnerable individuals [104]. Similarly, the Cincinnati Children’s Hospital Medical Center (CCHMC) developed the Youth Council for Suicide Prevention (YCSP) which has employed youth participatory action researchers to engage young people in critical reflection and action around the issue of adolescent suicide in Cincinnati, Ohio [105]. Through these efforts to include youth in the research and dissemination on this topic, youth developed a suicide prevention framework to aid in guiding suicide prevention activities that respond to contextualized youth needs [105].

Finally, specific policies and funding commitments should be made to support the production and availability of more comprehensive reporting of county, statewide, and national initiatives on suicidality and NSSI. These efforts have been supported by calls to action around improved data collection, increased funding for improved data infrastructures, timely assessments, universal documentation for emergency room visits, and coordination across jurisdictions [27]. With more comprehensive reporting on the resources and outputs of these initiatives, researchers can identify where resources are sparse across counties, communities, and states, and make efforts to fill these gaps.

## Figures and Tables

**Table 1:** Sources used for this narrative review.

University library databases searching for texts on suicidality, non-suicidal self-injury, and youth in the United States. Google Scholar searches for texts on suicidality, non-suicidal self-injury, and youth in the United States. Centers for Disease Control and Prevention WISQARS™ interactive, online database of fatal injury and leading causes of death. Attendance meetings, conference presentations, and webinars in the content area. Hand searches of the references of the retrieved literature.

**Table 2:** Measures of Youth Suicidal Ideation and Non-Suicidal Self-Injury.

Scales	Construct	Items	Response Format	Scoring	Reliability
Paykel’s Suicide Scale (PSS)	Suicidal ideation	5	Dichotomous	Sum scores	α = 0.93
Modified Scale for Suicidal Ideation (MSSI)	Suicidal ideation	18	4-point scale	Sum scores	α = 0.94
Suicidal Ideation Questionnaire (SIQ)	Suicidal ideation	30	7-point scale	Sum scores	r = 0.97
Beck Scale for Suicidal Ideation (BSSI)	Suicidal ideation	19	3-point scale	Sum scores	α = 0.89
Suicidal Behaviors Questionnaire (SBQ)	Suicidal ideation	34			
Positive and Negative Suicide Ideation (PANSI-PI/NSI)	Suicidal ideation	14	5-point scale	Sum scores	α = 0.81–0.94
Suicidal Behaviors Questionnaire—Revised (SBQ-R)	Suicidality	4	Mixed ^a^	Sum scores	α = 0.76–0.88
Suicide Probability Scale (SPS)	Suicidal ideation	36	4-point scale	Sum scores	α = 0.93
Columbia Suicide Severity Rating (C-SSRS)	Suicidality	23	Mixed ^a^	Sum scores of continuous response options	(α = 0.95)
Clinician-Administered Non-Suicidal Self Injury Disorder Index (CANDI)	Non-suicidal self-injury	6	Mixed ^a^	Sum scores of continuous response options	(α = 0.71)
Alexian Brothers Assessment of Self-Injury Scale (ABASI)	Non-suicidal self-injury severity	28	Mixed ^a^	Sum scores of continuous response options	(α = 0.75)
Non-Suicidal Self-Injury Disorder Scale (NSSIDS)	Non-suicidal self-injury disorder	16	Mixed ^a^	Sum scores of continuous response options. Responses greater than four indicate respondent meets criteria.	(α = 0.76)

Note: Measures extracted from [23,24]. ^a^ Mixed response format may include a combination of dichotomous and continuous response options as well as questions pertaining to a history of suicidality, frequency, and severity. Mixed response may also include open-ended questions about (1) type or description of ideation, (2) plans, and (3) failed, aborted, or interrupted attempts.

**Table 3:** Rates of Suicide and Non-Suicidal Self-Injury Across Youth by Race/Ethnicity and Gender.

Race/Ethnicity	Suicide Rates—Boys	Suicide Rates—Girls	Cause of Death Ranking	Primary Cause(s) of Death	Non-Suicidal Self-Injury Rates
American Indian or Alaskan Native	26.22 per 100,000 ^a^	12.21 per 100,000 ^a^	#1 10–14 years old ^b^ #2 15–24 years old ^b^	Suffocation ^b^	20.79 per 100,000 ^c^
Non-Hispanic White	13.19 per 100,000 ^a^	2.87 per 100,000 ^a^	#2 10–14 years old ^b^ #2 15–24 years old ^b^	Suffocation ^b^ Firearms ^b^	17.71 per 100,000 ^c^
Non-Hispanic Black	7.33 per 100,000 ^a^	2.65 per 100,000 ^a^	#4 10–14 years old ^b^ #3 15–24 years old ^b^	Suffocation ^b^ Firearms ^b^	12.10 per 100,000 ^c^
Hispanic	7.24 per 100,000 ^a^	2.84 per 100,000 ^a^	#3 10–14 years old ^b^ #2 15–24 years old ^b^	Suffocation ^b^	19.19 per 100,000 ^c^
Asian American or Pacific Islander	6.74 per 100,000 ^a^	3.63 per 100,000 ^a^	#1 10–14 years old ^b^ #2 15–24 years old ^b^	Suffocation ^b^	14.98 per 100,000 ^c^

Note. Superscripts represent citations: ^a^ [34]; ^b^ [53]; ^c^ [54].
